# Chyromyidae (Diptera, Acalyptrata) of Turkey

**DOI:** 10.3897/zookeys.872.35378

**Published:** 2019-08-20

**Authors:** M.J. Ebejer, M. Barták

**Affiliations:** 1 Entomology Section, Department of Natural Sciences, National Museum of Wales, Cathays Park, Cardiff, UK National Museum of Wales Cardiff United Kingdom; 2 Department of Zoology and Fisheries, Faculty of Agrobiology, Food and Natural Resources, Czech University of Life Sciences Prague, Kamýcká 129, 165 00 Praha-Suchdol, Czech Republi Czech University of Life Sciences Prague Czech Republic

**Keywords:** Brachycera, faunistics, Schizophora, West Palaearctic

## Abstract

The Chyromyidae of Turkey are reviewed and all 15 species known from the country are listed. The following are new records: *Chyromya miladae* Andersson, 1976, *Gymnochiromyia inermis* (Collin, 1933), *Aphaniosoma approximatum* Becker, 1903, *A. micromacro* Carles-Tolrá, 2001, *A. propinquans* Collin, 1949 and *A. proximum* Ebejer, 1998.

## Introduction

The Chyromyidae is a poorly recorded family from several countries around the Mediterranean despite this family of acalyptrate Diptera being very diverse in this region. Until recently this family was poorly represented in most collections. Ebejer (2009) detailed what little is known about the biology and ecology of these flies.

A review of *Aphaniosoma* Becker, 1903 included some records and descriptions of four new species of this genus from Turkey (Ebejer 1998a). Each of two papers on *Gymnochiromyia* Hendel, 1933 added one new record of species in this genus (Ebejer 1998b, 2010). No other literature records of Chyromyidae from Turkey are known to us. In particular, it must be noted that there are no Chyromyidae known from the north, central, and eastern provinces of the country. There is likely to be an interesting diversity probably enriched by species from Central Asia from where many species were described (Ebejer 2006). Species of *Aphaniosoma* have been found at high altitudes in Central Asia where the climatic and ecological conditions are rather different from those typical of the Mediterranean, but to some extent are similar to those in mountainous eastern Turkey. In this paper we collate what is known about Turkish Chyromyidae and add new records.

## Materials and methods

**Figure 1.**
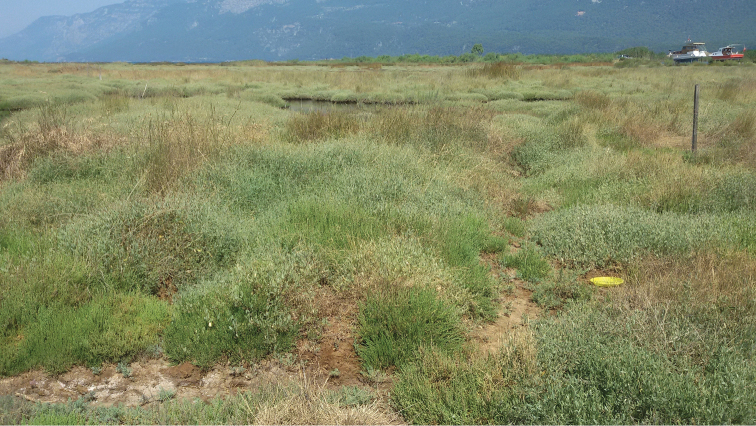
Wetland in Akyaka, SW Turkey. The locality inhabited by the maximum number of Chyromyidae, ten species.

Most of the new material presented in this paper originated from Muğla Province (Akyaka, Toparlar, Dalyan, and Muğla university campus). Akyaka (Fig. 1) represents a typical habitat for *Aphaniosoma*. It is a wet salty meadow, partly influenced by tides. However, this locality was severely destroyed after 2015 by agricultural and recreational development. The saline meadow at Dalyan remains relatively unaffected (farm and orchard sites are situated close to this wetland). A small proportion of specimens originates from Aydın Province (Çine) where specimens were collected from the banks of a river flowing out of a dam, ensuring year-round flow of water. Many other rivers in southern Turkey dry out in summer, supporting only very poor dipteran populations.

All species are listed below in alphabetical order under each subfamily and genus. Additional new data for each species are included where these are available and new records for Turkey are indicated. The material that has been examined for this paper was collected by M. Barták and Š. Kubík, unless otherwise stated, and by using yellow water pan traps (PN), hand held sweep nets (SW), and Malaise traps (MT). Depositories of specimens are in M. Barták collection, Czech University of Life Sciences, Prague, unless otherwise stated and given in parenthesis at the end of each data entry thus: **MJE** = M.J. Ebejer collection, Cowbridge, UK; **SMOC** = Silesian Museum Opava, Czech Republic.

## Results

### Aphaniosominae

#### *Aphaniosoma* Becker, 1903

##### *Aphaniosoma approximatum* Becker, 1903

**Material examined.** 7♂♂, Muğla Province, Akyaka, river bank, salty meadow, 37°03'16"N, 28°19'57"E, 16–27.v.2011; 1♂, same data (MJE).

This is the first confirmed record in the Mediterranean north of Egypt from where it was described. It is known from most of the Arabian Peninsula. Old records from Southern Europe and the Mediterranean are likely to refer to other similar species.

##### *Aphaniosoma brevivittatum* Ebejer, 1995

Recorded by Ebejer (1998a).

**Material examined.** 1♂, Akyaka, pasture, 6 m, SW, 37°03'19"N, 28°20'07"E, 28.iv–8.v.2013; 5♂♂, Akyaka, salty meadow, SW+PT, 37°12'45"N, 28°27'42"E, 28.iv–9.v.2013, (1♂, MJE); 2♂♂, Akyaka, pasture, 4 m, 37°03'09"N, 28°20'17"E, 8–14.ix.2014; 1♂1♀, Akyaka, pasture, 4 m, 37°03'09"N, 28°20'17"E, 13–14.ix.2014; 2♂♂, Akyaka, salty meadow, 2 m, 37°01'49"N, 28°20'01"E, 22.vi–1.vii.2015.

##### *Aphaniosoma claridgei* Ebejer, 1995

Recorded by Ebejer (1998a).

**Material examined.** 9♂♂7♀♀, Akyaka, river bank, salty meadow, 37°03'16"N, 28°19'57"E, 16–27.v.2011; 4♂♂1♀, Akyaka, pasture, 4 m, 37°03'08.9"N, 28°20'17.4"E, 16–22.ix.2012; 1♂, Akyaka, pasture, 4 m, 37°03'09"N, 28°20'17"E, 23–27.ix.2012; 1♀, Akyaka, salty meadow, SW+PT, 37°12'45"N, 28°27'42"E, 28.iv–9.v.2013; 2♀♀, Akyaka, salty meadow, SW+PT, 37°02'53"N, 28°19'39"E, 28.iv–9.v.2013; 3♂♂, Akyaka, salty meadow, 2 m, 37°01'49"N, 28°20'01"E, 22.vi–1.vii.2015; 1♀, 8 km S of Çine, river bank, 68 m, 37°32'34"N, 28°03'46"E, 29.iv–i.v.2016.

A very common and abundant species described from Greece but found in most of the countries around the Mediterranean.

##### *Aphaniosoma impudens* Ebejer, 1998

Described from Turkey. No new material has been examined.

##### *Aphaniosoma melitense* Ebejer, 1993

Recorded by Ebejer (1998a).

**Material examined.** 2♂♂4♀♀, Antalya, Manavgat, 3.5 km S, Titreyen lake, 1 m, 36°45'25"N, 31°27'19"E, 15.v.2011, J. Roháček (SMOC); 40♂♂12♀♀, Akyaka, river bank, salty meadow, 37°03'16"N, 28°19'57"E, 16–27.v.2011; 15♂♂6♀♀, Akyaka, pasture, 4 m, 37°03'08.9"N, 28°20'17.4"E, 16–22.ix.2012; 1♂1♀, Akyaka, salty meadow, 2 m, 37°03'N, 28°20'E, 23–27.ix.2012; 4♂♂2♀♀, Akyaka, pasture, 4 m, 37°03'09"N, 28°20'17"E, 23–27.ix.2012; 1♂, Akyaka, pasture, 6 m, SW, 37°03'19"N, 28°20'07"E, 28.iv–8.v.2013; 8♂♂, Akyaka, salty meadow, SW+PT, 37°12'45"N, 28°27'42"E, 28.iv–9.v.2013; 1♂1♀, Akyaka, salty meadow, SW+PT, 37°02'53"N, 28°19'39"E, 28.iv–9.v.2013; 3♂♂, Akyaka, pasture, 4 m, 37°03'09"N, 28°20'17"E, 8–14.ix.2014; 5♂♂3♀♀, Dalyan, orchard, 4 m, 36°49'37"N, 28°39'39"E, 11.ix.2014; 7♂♂1♀, Toparlar, lowland forest, 8 m, 36°59'27"N, 28°38'50"E, 11.ix.2014; 3♂♂, Akyaka, pasture, 4 m, YPWT, 37°03'09"N, 28°20'17"E, 13–14.ix.2014; 1♂, 8 km S of Çine, river bank, 68 m, 37°32'34"N, 28°03'46"E, 28–30.vi.2015; 12♂♂1♀, Akyaka, salty meadow, 2 m, 37°01'49"N, 28°20'01"E, 22.vi–1.vii.2015; 1♂2♀♀, Mugla, 730 m, university campus, MT, 37°09'19"N, 28°20'07"E, 5–19.viii.2015, H. Kavak; 1♂, Dalyan, farm, MT, 1 m, 36°48'54"N, 28°39'04"E, 8–20.viii.2015, Dursun; 1♀, Akyaka, 40 m, forest, SW, 37°03'19"N, 28°19'36"E, 26.iv.2016.

This common and often abundant species was described from Malta. It is known from most of the countries around the Mediterranean and reaches Britain.

##### *Aphaniosoma micromacro* Carles-Tolrá, 2001

**Material examined.** 1♂, Akyaka, pasture, 6 m, SW, 37°03'19"N, 28°20'07"E, 28.iv–8.v.2013.

This species was described from Spain. New record for Turkey.

##### *Aphaniosoma necopinatum* Ebejer, 1998

Described from Turkey. No new material has been examined.

##### *Aphaniosoma propinquans* Collin, 1949

**Material examined.** 1♂1♀, Akyaka, river bank, salty meadow, 37°03'16"N, 28°19'57"E, 16–27.v.2011; 1♀, Akyaka, pasture, 6 m, SW, 37°03'19"N, 28°20'07"E, 28.iv–8.v.2013; 1♂, Akyaka, salty meadow, 37°02'53"N, 28°19'39"E, 28.iv–9.v.2013; 1♀, Akyaka, salty meadow, 37°12'45"N, 28°27'42"E, 28.iv–9.v.2013; 1♂1♀, 8 km S of Çine, river bank, 68 m, 37°32'34"N, 28°03'46"E, 28–30.vi.2015.

Described from Britain, this is a widespread and fairly common species. New record for Turkey.

##### *Aphaniosoma proximum* Ebejer, 1998

**Material examined.** 1♂, Akyaka, pasture, 4 m, 37°03'08.9"N, 28°20'17.4"E, 16–22.ix.2012; 5♀♀, Akyaka, salty meadow, 2 m, 37°03'N, 28°20'E, 23–27.ix.2012; 1♀, Akyaka, salty meadow, SW+PT, 37°12'45"N, 28°27'42"E, 28.iv–9.v.2013; 1♂, Akyaka, pasture, 4 m, 37°03'09"N, 28°20'17"E, 8–14.ix.2014; 5♂♂1♀, Akyaka, salty meadow, 2 m, 37°01'49"N, 28°20'01"E, 22.vi–1.vii.2015.

A common and widespread species around the Mediterranean, extending as far as the United Arab Emirates. Sometimes found in large numbers. A new record for Turkey.

##### *Aphaniosoma scutellaris* Ebejer, 1998

Described from Turkey, it has also been recorded from Germany.

##### *Aphaniosoma verecundum* Ebejer, 1998

Described from Turkey.

**Material examined.** 1♀, Akyaka, pasture, 4 m, 37°03'08.9"N, 28°20'17.4"E, 16–22.ix.2012; 1♂2♀♀, Akyaka, salty meadow, 2 m, 37°01'49"N, 28°20'01"E, 22.vi–1.vii.2015; 1♀, Akyaka, salty meadow, 2 m, PT, 37°01'52"N, 28°20'00"E, 27.iv–1.v.2016.

### Chyromyinae

#### *Chyromya* Robineau-Desvoidy, 1830

##### *Chyromya miladae* Andersson, 1976

**Material examined.** 1♀, Toparlar, lowland wood, 8 m, 36°59'27"N, 28°38'50"E, 22–24.vi.2015.

This species was described from the Czech Republic but it has been found in Britain, Switzerland, and Germany. A new record for Turkey.

#### *Gymnochiromyia* Hendel, 1933

##### *Gymnochiromyia flavella* (Zetterstedt, 1848)

Recorded by Ebejer (2010).

**Material examined.** 1♀, Antalya, Manavgat, 4.4 km S, Manavgat rivershore, 1 m, 36°45'01"N, 31°28'03"E, 15.v.2011, J. Roháček (SMOC); 1♀, 8 km S of Çine, river bank, 68 m, 37°32'34"N, 28°03'46"E, 29.iv–i.v.2016.

A common and widespread species throughout Europe extending from Scandinavia to North Africa.

##### *Gymnochiromyia inermis* (Collin, 1933)

**Material examined.** 1♀, Akyaka, river bank, salty meadow, 37°03'16"N, 28°19'57"E, 16–27.v.2011.

This species is a new record for Turkey. There are two other very similar species known from Israel and Lebanon. What is somewhat unusual for this record is the locality where it was found. These three species of *Gymnochiromyia* are most frequently encountered, though not exclusively so, in open woodland dominated by *Quercus*.

##### *Gymnochiromyia mihalyii* Soós, 1979

Recorded by Ebejer (1998b).

**Material examined.** 2♀♀, Akyaka, pasture, 6 m, SW, 37°03'19"N, 28°20'07"E, 28.iv–8.v.2013; 1♂, Akyaka, salty meadow, 37°12'45"N, 28°27'42"E, 28.iv–9.v.2013; 1♀, Akyaka, salty meadow, 37°02'53"N, 28°19'39"E, 28.iv–9.v.2013; 1♂, Akyaka, salty meadow, 2 m, 37°01'49"N, 28°20'01"E, 22.vi–1.vii.2015.

A widespread species in Europe and the Mediterranean, though not as common as *G. flavella*.

## Discussion

We list one species of *Chyromya* (a new record), three of *Gymnochiromyia* (two new records), and 11 of *Aphaniosoma* (three new records). Although we found no literature records of *Chyromya flava* (Linnaeus, 1758) this very widespread species certainly occurs in Turkey. *Chyromya britannica* Gibbs, 2007 was described from Britain and later found in France, but even this species is more widespread than previously thought. MJE has seen a specimen from Slovenia collected in 1958 and housed among unsorted material in the Natural History Museum, London, UK. Thus, its distribution may be more widespread than originally thought and it may also occur in Turkey. With regards to *Aphaniosoma*, given the numerous species present in the eastern part of the Mediterranean, the many undescribed species known to MJE, and the fact that eastern Turkey has not been investigated for this family, it is very likely that the fauna is currently very under represented. We estimate that approximately 30 species should occur in this country.
